# miRiadne: a web tool for consistent integration of miRNA nomenclature

**DOI:** 10.1093/nar/gkv381

**Published:** 2015-04-20

**Authors:** Raoul J. P. Bonnal, Riccardo L. Rossi, Donatella Carpi, Valeria Ranzani, Sergio Abrignani, Massimiliano Pagani

**Affiliations:** 1 Istituto Nazionale Genetica Molecolare ‘Romeo ed Enrica Invernizzi’, Via F. Sforza 35, 20122 Milan, Italy; 2 Department of Medical Biotechnology and Translational Medicine, Università degli Studi di Milano, Via Festa del Perdono 7, 20122 Milano, Italy

## Abstract

The miRBase is the official miRNA repository which keeps the annotation updated on newly discovered miRNAs: it is also used as a reference for the design of miRNA profiling platforms. Nomenclature ambiguities generated by loosely updated platforms and design errors lead to incompatibilities among platforms, even from the same vendor. Published miRNA lists are thus generated with different profiling platforms that refer to diverse and not updated annotations. This greatly compromises searches, comparisons and analyses that rely on miRNA names only without taking into account the mature sequences, which is particularly critic when such analyses are carried over automatically. In this paper we introduce miRiadne, a web tool to harmonize miRNA nomenclature, which takes into account the original miRBase versions from 10 up to 21, and annotations of 40 common profiling platforms from nine brands that we manually curated. miRiadne uses the miRNA mature sequence to link miRBase versions and/or platforms to prevent nomenclature ambiguities. miRiadne was designed to simplify and support biologists and bioinformaticians in re-annotating their own miRNA lists and/or data sets. As Ariadne helped Theseus in escaping the mythological maze, miRiadne will help the miRNA researcher in escaping the nomenclature maze. miRiadne is freely accessible from the URL http://www.miriadne.org.

## INTRODUCTION

MicroRNAs (miRNAs) are short endogenous non-coding RNAs varying in length from 19 to 25 nucleotides. They inhibit expression of their cognate target genes by post transcriptional regulation (1), playing a relevant role in a vast array of molecular and cellular processes (2). miRNAs have been shown to be involved in several diseases, such as type 2 diabetes, cardiovascular disease and cancer (3,4), to be key regulators of gene expression and differentiation (5,6) and they have been exploited as circulating biomarkers both in health and disease (7). The first miRNA was identified in *Caenorhabditis elegans* in 1993 (8,9), while the name ‘miRNA’ was introduced almost a decade later in 2001 (10). Subsequently miRNA research gained momentum and an organic naming system was proposed (11), then implemented into a curated and regularly updated repository largely adopted by the scientific community: the miRBase (12). miRBase collects precursor and mature miRNA sequences and is now the authoritative reference for miRNA nomenclature constantly addressing the increasing complexity using and updating suffixes of mature miRNA names.

Microarray and RTqPCR-based expression profiling methods of mature miRNAs have become increasingly common in recent years establishing themselves as methods of election for miRNA expression studies. Next-generation sequencing technologies are now pushing forward the discovery of new sequences significantly increasing the number of known miRNAs in each specific organism (13). The correct categorization and collection of mature miRNAs into consistently annotated catalogs is thus an instrumental process for managing and integrating miRNA expression data sets. Unfortunately the fast changing rate of miRNA nomenclature generates confusion in miRNA research that is acknowledged by the scientific community: this problem has been addressed by the development of webservers that allows to browse across versions of the miRBase such as the ‘miRBase Tracker’ (14) and ‘miRSystem’ (15). While the first allows to browse miRNA annotations according to the miRBase, the second is mainly devoted to the characterization of miRNA targets and contains a feature used to convert miRNAs names; both of them use the miRBase only as underlying source of heterogeneous annotations. miRBase also provides a list of previous names of its entries, nevertheless none of these resources deal with sequences generated by the profiling platforms. The highly detrimental miRNA nomenclature maze is not only due to the evolving annotations, but also—and somehow mainly—due to the use of heterogeneously annotated profiling data sets generated by diverse and not updated detection platforms: this often impairs the straightforward integration of different data sets to deliver meta analyses that could be beneficial to miRNA research. On these premises we decided to implement an application specifically devoted to the re-annotation of miRNA signatures and profiling data sets, natively embedding the annotation and probe sequences of 40 common profiling platforms (27 human and 13 rodents) from nine brands integrated with the annotations of miRBase from version 10 to the most recent.

Our webserver application, miRiadne, goes beyond browsing miRNA nomenclature across miRBase versions, by offering translation and updating of miRNA namelists and data sets annotations enforcing consistency between miRNA name and their mature sequences. The novelty of our application is the curation of experimental profiling platforms’ annotations checking for the consistency between miRNA names used by these platforms and their probes’ sequences. Such consistency is a prerequisite that would make meta analysis of miRNA expression data sets easier. While the community is moving toward higher quality standards for miRNA repositories (13) and efforts are made to re-annotate miRNA entries with data from next-generation sequencing experiments (16), heterogeneous and inconsistent miRNA profile nomenclature will remain in the literature affecting future meta analyses; moreover, many detection platforms that adopt different standards are still in use thus generating more inconsistencies. Therefore miRiadne aims at improving interoperability among miRNA data sets and at exploiting all the information contained in already produced miRNA profiling data without any loss of knowledge.

## IMPLEMENTATION

The tool was implemented as a web application written in Ruby on Rails. miRBase data from version 10 to 21 were downloaded (in the form of the officially released txt files), from the miRBase FTP server and reorganized in a SQLite database. Probes’ Annotation Information Files from 40 different detection platforms of nine vendors (Supplementary Tables S1 and S2 and Supplementary Figures S1 and S2) were retrieved and used in the application as reference for the miRNA name to mature sequence correspondence of each single probe (Supplementary Figure S3). Probes annotation files were stored in the SQLite database. miRiadne is fully compliant with HTML5 and using Twitter Bootstrap framework provides a responsive web site for variable desktop sizes and mobile devices.

## FEATURES AND INPUTS

**Figure 1.**
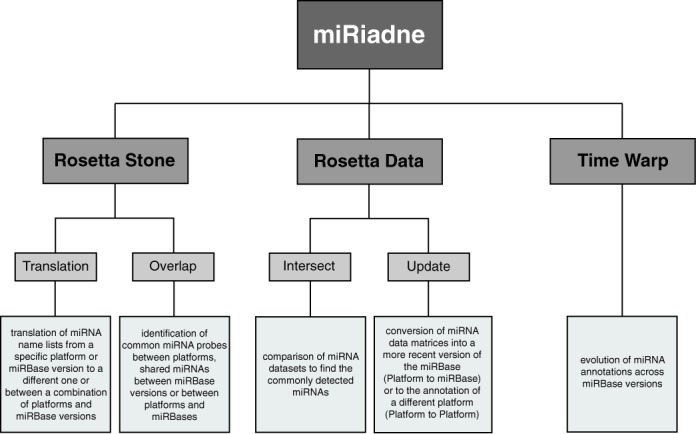
miRiadne main features. The five miRiadne's features grouped in two main classes: the first class includes the four Rosetta features used to translate miRNA names (Rosetta Stone functions) and data sets (Rosetta Data functions) produced by miRNAs detection platforms: they are the Translation, Overlap, Intersection and Update functions. The second main feature is the Time Warp that allows one to browse any miRNA name or name lists evolution across miRBase versions, in any species.

miRiadne application presents two main features: the first one includes four slightly different methods, collectively referred to as ‘Rosetta functions’, and allows the user to translate miRNA names from diverse sources into more established and controlled ones: they are the functions ‘Translation, and Overlap’ (in the ‘Rosetta Stone’ menu) and the functions ‘Update and Intersect’ (in the ‘Rosetta Data’ menu). While the first two work on miRNA names only, the third and fourth accept also whole miRNA expression data sets. The second main feature is the ‘Time Warp’ function that allows the user to browse the evolution of miRNA annotations across miRBase versions and serves as a standalone function or as a substantial consistency crosscheck for any miRNA list whose annotation has been translated or converted with the Rosetta functions (Figure 1).

Data can be entered into the miRiadne application in a few different ways: all miRiadne's functions are accessed by a main ‘input data window’ where miRNA names can be typed one by one or they can be pasted from external documents; either typed or pasted, miRNA names must be separated by a space or a new line. Lists can also be imported directly dragging files containing the query miRNA list into the data window (files being in tab delimited or comma separated values format).

### Rosetta Stone—Translation

This feature allows to translate miRNA name lists from a specific platform's annotation to a different one, from a specific miRBase version to a different one or between a combination of platform and miRBase versions: any of the four possible paired combinations among platforms and miRBases is allowed and can be selected choosing one of the four buttons below the data input window (Supplementary Figure S4). The user can enter a miRNA name or name list as it appears in a given vendor platform (among the platforms implemented as shown in Supplementary Tables S1 and S2) or as reported in a list referring to a specific miRBase version, and obtain a translation either (i) into names of a different, ‘destination’ platform, or (ii) into names according to a different, usually more recent, miRBase version. After entering the miRNA names in the specific field and choosing one of the available conversions, a contextual menu appears offering the user the possibility to specify sources and destination (i.e. from which specific profiling platform to which miRBase version); then a simple click to the ‘convert’ button results in the elaboration and production of the Result Table. Submission of such a query will result in a converted list of names, with graphical evidence for miRNAs that cannot be translated due to different reasons (a different background for dead, changed or not present miRNAs or for miRNAs with multiple names). The Result Table can also be downloaded as csv file.

### Rosetta Stone—Overlap

The ‘Overlap’ function does not need any input query file because it is used to find and highlight the miRNAs in common between two different detection platforms (i.e. present on both platforms with the same mature sequence regardless names variations), between two miRBase versions or between platforms and miRBase versions. The first case (Platform versus Platform) can be used to evaluate the potential detection ability of different platforms (Supplementary Figure S5), the second case (Platform versus miRBase) is useful to evaluate the miRBase coverage of different detection platforms; finally the third case (miRBase versus miRBase) shows which miRNAs are conserved, in terms of sequence conservation between two different miRBase versions even after any annotation change. This function directly interrogates the integrated database populated by the miRBase versions from 10 to the most recent and the annotations files from the detection platforms considered.

### Rosetta Data—Update

This function is a particular form of annotation translation in which annotation of miRNA data matrices (i.e. miRNA lists with expression values for a number of samples in tabular format) can be updated to a more recent version of the miRBase (Platform to miRBase), or converted to the annotation of a different detection platform (Platform to Platform), carrying over the corresponding data (expression values) from the original data set (Supplementary Figure S6). This function can be used to convert miRNA profiling data sets previously generated with different platforms or referring to different miRBase versions prior to a new analysis or just for comparison with more updated data sets. The input data should be in tabular form, where rows are relative to miRNAs and columns to samples: thus the first column contains the miRNA list used as query list, the first row (the header) reports the samples’ id and all other columns the expression data that will be carried over in the ‘Data’ column of the Result Table.

Annotations (miRNA names) of the original data set are reported in the Result Table side by side with the updated annotations (name and mature sequence) and the corresponding expression values in the original data set are carried over in the ‘Data’ column. The updated data matrix can be downloaded as a csv file, downloaded data values can be displayed in separate columns too, thus allowing further elaboration and/or analyses.

In case input query miRNA names cannot be translated or are not present any more in the updated annotation set: a warning is displayed. A detailed list of miRNAs that cannot be found in the destination annotation (either a more recent miRBase version or a different platform) is reported shaded in red at the bottom of the table. The single hits that may have changed, dropped out or associated with multiple names are highlighted with a yellow (missing, dead or changes) or blue (multiple names) shading in the table. Non-miRNA annotations such as those from control probes, non miRNA-probes or endogenous controls (i.e. any annotation which is by definition not included in the miRBase) cannot be recognized and thus will trigger the above mentioned warning.

### Rosetta Data—Intersection

This feature allows finding common miRNAs from two profile data sets (data matrices with miRNA expression values/rows in a number of samples/columns) regardless any change that could have occurred to the miRNA names. This function is useful when one needs to obtain a new data set from two human miRNA data sets generated by two different profiling platforms which contains common miRNAs only. The newly generated data set will be populated with all the miRNAs present in both original data sets according to the mature sequence (i.e. that have the same sequence), thus allowing to perform downstream analysis without the risk of missing any information (Supplementary Figure S7). This feature also allows the carryover of the expression values (only of the miRNAs shared between the two data sets) and the input format of the query data set tables is the same as the one previously described for the ‘Update’ function.

### Time Warp

This is the other main function of the miRiadne application: it allows to track miRNA names evolution (name changes, survival and/or drop outs) through the miRBase versions from number 10 to the most current one. Name changes, new miRNA entries and miRNAs deleted from the miRBase are taken into consideration. Mature sequence for each entry is always appended.

User can input one or more miRNA names, directly typing them or uploading (drag and drop method) a text file with the names list. The search can be started from a selected miRBase version, or, alternatively, the default search that spans all miRBases in miRiadne (from version 10 to the latest miRBase) can be performed. Searches can be stringent (default) or relaxed: in the first case search will retrieve only miRNAs with exactly the same name as typed, in the second case all miRNA names containing the search string will be retrieved. The *Homo sapiens* is set as the default species but if the species is not selected the search will be performed on the entire miRBase and results will comprise all species in the miRBase. As an additional function it is also possible to display the profiling platforms implemented in miRiadne that can detect the searched miRNA(s). Matching miRNA(s) are displayed in a result table with relative IDs (MI and MIMAT), names, sequences, strand and length: a graphical indicator shows if that miRNA is stable, if it was deleted, at which miRBase version the searched miRNA was introduced (the name is introduced) or a name change occurred (new name displayed) (Supplementary Figure S8).

## DISCUSSION

Detection methods based on hybridization technologies such as microarrays or RTqPCR that exploit designed probes to detect mature miRNA expression are widely used and their probesets refer to and are designed on different miRBase releases. Unfortunately, miRNA probe lists in the detection platforms sometime are not explicitly associated with probe sequences and often the miRBase unique identifier (the so-called MIMAT id) is often not reported. Moreover, a substantial portion of publications on human miRNAs (more than half, as shown in a survey of 100 recent papers (14)) relies on miRNA names without disclosing sequences of investigated miRNAs, thus contributing to annotation uncertainty in the literature.

Other tools have partially overlapping functions with parts of miRiadne: for instance the miRBase Tracker (14) is redundant with the miRiadne's Time Warp function and miRBase itself provides a list of previous names of miRNA sequences. Nevertheless, miRiadne offers a novel profiling platform-centric approach which protects from the confusion generated by names used in publications and experimental resources that have become out-of-date with the official names in the current version of the miRBase. While the miRBase is regularly updated and miRNA sequences can be renamed, or even deleted or added, the commercial probesets do not undergo a regular re-annotation (exceptions exist) thus they are usually not updated. The result is high heterogeneity in names usage when referring to list of expressed, differential expressed or selected miRNAs. Lower costs and availability of high-throughput technologies in the miRNA profiling field generated so far a body of literature suffering from a relevant degree of nomenclature inconsistencies; moreover, the lack of standard guidelines for data profiling hampers the possibility to take advantage from the wide variety of tissues and conditions in which miRNAs expression has been investigated. miRBase recently began an effort of additional annotation producing a high confidence side version of the miRBase using next-generation sequencing data (13); this is an admirable intent but, as stated by miRBase curators themselves, miRNA names remain ‘entirely unsuitable to encode information about complex sequence relationships’ (17).

**Figure 2.**
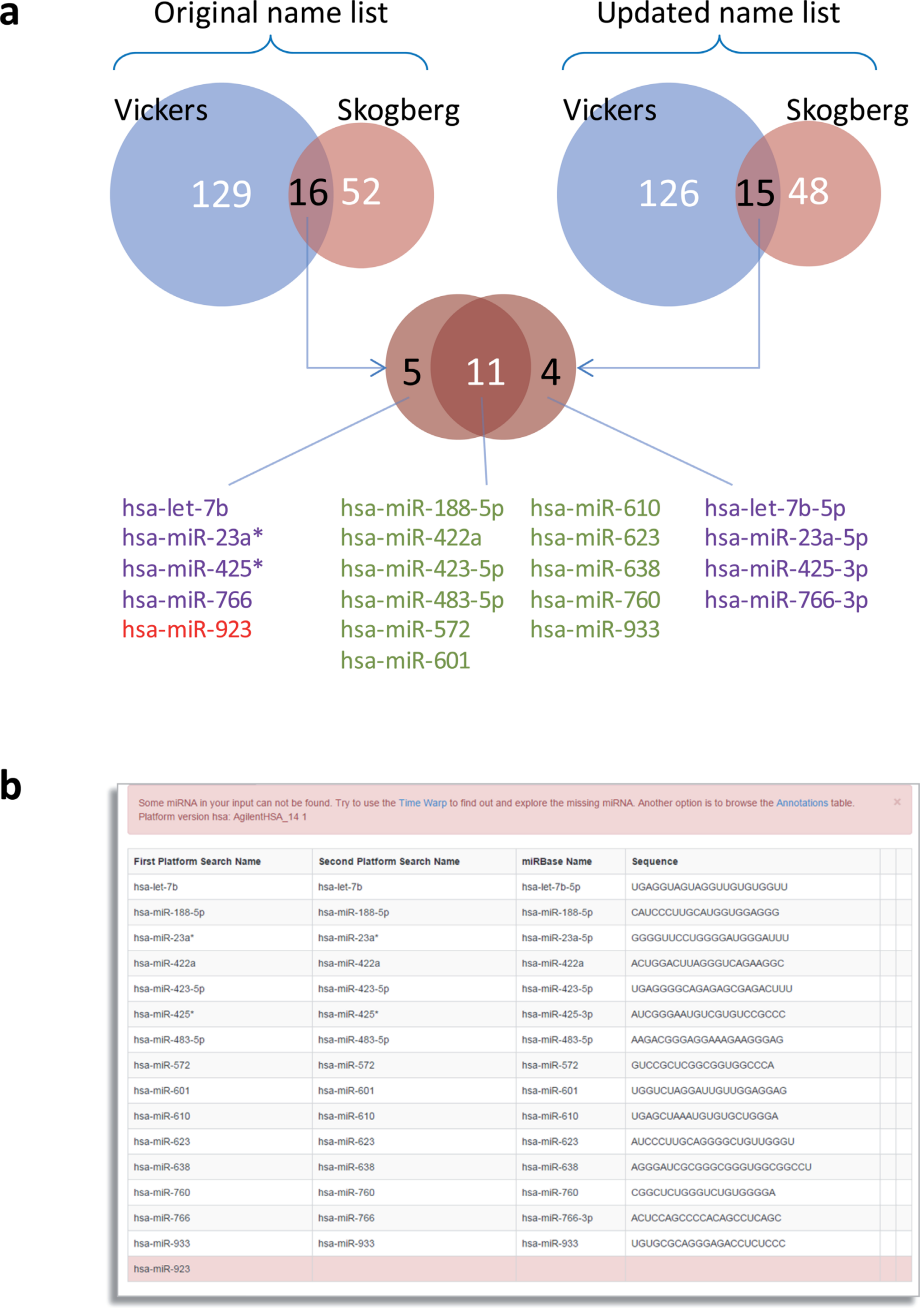
Name-driven miRNA list comparison. (**a**) Overlap of miRNA signatures from two different papers (18,19). Overlap between miRNA names as they are reported in the papers (Venn diagram on the left) shows 16 commonly detected miRNAs, while overlap between miRNA signatures with updated annotations (Venn diagram on the right) shows 15 common miRNAs. Further overlap between the two commonly detected miRNAs uncovers that only 11 miRNAs are not affected by inconsistent annotations between the two papers and are correctly included in the comparison in both cases, and one miRNA (hsa-miR-923, in red) is not present in the updated list. (**b**) The 15 miRNAs commonly detected by the two works in the result table obtained with the ‘Rosetta Intersect’ function. Names are automatically updated to latest miRBase version, mature sequences appended and retired miRNAs (such as hsa-miR-923) highlighted and excluded. Destiny of retired miRNAs can be further investigated with the Time Warp function.

Thus evolving nomenclature leads to inconsistencies, and as a consequence, name-driven comparisons of miRNA lists (such as signatures) from diverse sources (papers, databases) can be risky, and higher meta-analyses virtually impossible. As an example, we extracted the miRNA signature from two recent papers about miRNAs carried by human exosomes (18,19) and compared them both ‘as they are’ originally reported and ‘after translation’ using the miRiadne ‘Intersect’ function. The two works used different platforms and, as expected, slightly different experimental conditions: nonetheless if miRNAs detected both in exosomes (18) and in HDL-associated exosomes (19) were searched for, performing a name based overlap analysis between the two original signatures, discrepancies not merely due to annotations would be found. The use of miRiadne allows to perform the signatures’ comparison much faster, with sequence consistency and uncovering four name changes and one retired miRNA (Figure 2); such differences would not have been detected by a mere manual comparison of miRNA names.

Even subtler changes can take place across miRBase versions, deriving from immediate replacement of dead entries when the biogenesis process is discovered and needs to be corrected; such a case is the one of hsa-miR-453 that being processed from the 5p arm of miR-323b from miRBase 15 onward became hsa-miR-325b-5p. Such changes would not be a problem for researchers focused on a few or a single miRNA, which can be individually followed in its evolution by the Time Warp function of miRiadne or other applications (14,15) but it is a major bottleneck when doing screening and automated data processing of profiling data: the use of a tool such as miRiadne will alleviate the difficulty in performing meta analyses with miRNA expression profiling data.

## SUPPLEMENTARY DATA

Supplementary Data are available at NAR Online.

SUPPLEMENTARY DATA
