# Supplementary material for: miRiadne: a web tool for consistent integration of miRNA nomenclature
Source: Nucleic Acids Res. 2015 Apr 20;43(Web Server issue):W487–92. doi: 10.1093/nar/gkv381 (PMC4489305; doi:10.1093/nar/gkv381)
Supplement: SUPPLEMENTARY DATA [file supp_43_W1_W487__index.html]

miRiadne: a web tool for consistent integration of miRNA nomenclature — miRiadne: a web tool for consistent integration of miRNA nomenclature — SUPPLEMENTARY DATA 

# miRiadne: a web tool for consistent integration of miRNA nomenclature

## SUPPLEMENTARY DATA

**Files in this Data Supplement:**

- SUPPLEMENTARY DATA
