# Supplementary material for: miRiadne: a web tool for consistent integration of miRNA nomenclature
Source: Nucleic Acids Res. 2015 Apr 20;43(Web Server issue):W487–92. doi: 10.1093/nar/gkv381 (PMC4489305; doi:10.1093/nar/gkv381)

Supplementary Material for

miRiadne: a web tool for consistent integration of miRNA nomenclature

SUPPLEMENTARY FIGURE AND TABLE CAPTIONS

Supplementary table 1. Human profiling platforms. Table of detection profiling platforms whose annotation information files were considered and that are used by the miRiadne translation engine. Columns of the table show, from left to right: brand and commercial name of platform, platform version (release), technology used (microarray, RTqPCR or beads counter); miRBase version to which the platform's annotations refer to, number of miRNAs detectable by the platform; platforms that disclose miRNA probes sequences, platform that do not disclose them (or for which a sequence retrieval from miRBase was necessary).

Supplementary figure 1. Number of human miRNAs per platform. Histogram showing number of human miRNAs detected by the 27 platforms taken into account by miRiadne. Control probes were excluded from the count.

Supplementary table 2. Rodent profiling platforms. Table of detection profiling platforms whose annotation information files were considered and that are used by the miRiadne translation engine. Columns of the table show, from left to right: brand and commercial name of platform, platform version (release), technology used (microarray, RTqPCR or beads counter); miRBase version to which the platform's annotations refer to, number of miRNAs detectable by the platform; platforms that disclose miRNA probes sequences, platform that do not disclose them (or for which a sequence retrieval from miRBase was necessary).

Supplementary figure 2. Number of rodent miRNAs per platform. Histogram showing number of mouse and rat miRNAs detected by the of the 13 platforms taken into account by miRiadne. Control probes were excluded from the count.

Supplementary figure 3. AIFs validation procedure. The figure shows the validation process of AIFs (Annotation Information Files, the probes' annotations supplied with each detection platform) before their inclusion into miRiadne database. If the probe/miRNA sequences were originally included in the supplied AIF they were taken into account and stored with miRNA names; if they were not, mature miRNA sequences were exrtacted from the miRBase version whose the specific platform refers to, then subjected by a manual curation to find any inconsistence between probe names and miRBase miRNA names.

Supplementary figure 4. Rosetta Stone - Translation. How to use the Rosetta translation function: select the function from menu (1-2); import the data either by typing it or dragging and dropping in the window a txt file (tab delimited or comma separated value) with the miRNA names list (3); select one of the four possible translation combination, in the screenshot the "from platform to miRBase" is selected (4); select the starting platform and the destination miRBase from the corresponding menus (5); run the conversion (6); review the result table (7). The results display the miRNA names searched, the names in the starting and destination queries (before and after translation), the alternative name (if present in the detection platform's annotation) and the mature sequence. Two cases in which a single miRNA has been annotated with more than one name (8) and one case of a retired miRNA (9) are shown (the result table in the screenshot is truncated).

Supplementary figure 5. Rosetta Stone - Overlap. How to use the Rosetta overlap function: this function does not need any input file since it is used to see overlap between platforms' probes and/or miRBase versions. Three options are available, overlap between different platforms, between different miRBase versions and between specific platforms and miRBase versions. Select the function from menu (1-2); choose one of the three options (3), select the two terms of comparison, either platforms and/or miRBases according to the option chosen (4-5); run the job (6) and review the result table (7). The result table contains all and only those miRNAs that, according to mature sequence identity, are contained in both terms of comparison.

Supplementary figure 6. Rosetta Data - Update. How to use the Rosetta update function: select the function from menu (1-2); import the data, the easier and safer way is to drag and drop in the window a txt file containing the miRNA expression dataset (tab delimited or comma separated value) with the miRNA names list in the first column (3); choose one of the two possible options, either to update the dataset from the generating platform to a specific miRBase version or to the annotation of another detection platform (4); specify the generating platform on the left and the landing translation on the right (5); run the translation job (6) and review the result table (7). The result table contains all and only those miRNAs that are present in the landing platform or miRBase (i.e. those miRNAs that can be updated given the chosen parameters), the mature sequence is displayed and the original data matrix is appended in the "Data" column. The result table can be downloaded (8) as a comma separated value file and quickly imported in spreadsheet format, the data column which contains space delimited data is also carried over and easily restored (9).

Supplementary figure 7. Rosetta Data - Intersect. How to use the Rosetta intersect function: select the function from menu (1-2); import the data either by typing it or dragging and dropping in the two windows a txt file (tab delimited or comma separated value) with the miRNA names list: a separate input window is used for the two miRNA lists or datasets that are going to be intersected. In the case of profiling datasets (with

expression values) or long miRNA lists the "drag and drop" method is the easier and safer (3-4); specify the first (5) and the second (6) platform, then submit the job (7) and review the result table (8). miRNAs present in both submitted datasets or lists are displayed in the result table, their names are updated to the ltest miRBase version and mature sequence is appended. Those miRNAs in the input that cannot be found are highlighted at the bottom of the table and a warning messade is display at the top of the table.

Supplementary figure 8. Time Warp. How to use the Time Warp function: select the function from menu (1); decide whether to perform a stringent search (default) or select the "Relax search" option to search for partial or incomplete matches; you can also decide to display the platforms able to detect the searched miRNAs, if any (2); the data can be imported either by typing it directly or dragging and dropping in the window a txt file (tab delimited or comma separated value) with the miRNA names list. For short miRNA lists, miRNA names can be typed either separated by a new line (carriage return) or separated by a comma (3). Select the miRNA version to be considered as the one where to start the analysis from, the default span is all miRBases in miRiadne, i.e. from version 10 to the latest (4); select the species, the default is Homo sapiens (5): if the "blank" option is explicitly selected, search is performed over all species in miRBase, and "Relax search" should be selected too in order to facilitate matching; run the job clicking "Search" (6) and review the result table (7). The result table displays searched miRNAs with their IDs, sequence, strand positioning and length. If no specific miRBase versions were selected the table display results from miRBase 10 to the latest and for each version a colored dot is used to indicate stability of the annotation (green dot) or retirement of the miRNA (red dot). Name changes are indicated with the new miRNA name in place of the dot. If a specific miRBase version was selected (miRBase 14 as shown in the screenshot) (8) Time Warp will start analysis from that version number and any miRNA occasionally retired before that version will not displayed (as it is the case of hsa-miR-801 in the figure) (9).

Supplementary Table 1

	Platform name	Release	Tecnology	miRBase	# miRNA	Seq by vendor	Seq by miRBase
Affymetrics	GeneChip miRNA Array	1	microarray	11	847	x	
	GeneChip miRNA Array	2	microarray	15	1105	x	
	GeneChip miRNA Array	3	microarray	17	1733	x	
	GeneChip miRNA Array	4	microarray	20	2578	x	
Agilent	Human miRNA Microarray	2	microarray	10.1	719		x
	Human miRNA Microarray	3	microarray	12	888		x
	Human miRNA Microarray	14	microarray	14	887		x
	Human miRNA Microarray	16	microarray	16	1205		x
	Human miRNA Microarray	18	microarray	18	1887		x
	Human miRNA Microarray	19	microarray	19	2006		x
LifeTech (Applied Biosystems)	TaqMan Array Human microRNA	2	RT-qPCR	10	664	x	x
	TaqMan Array Human microRNA	3	RT-qPCR	14	754	x	
	OpenArray Human microRNA	3	RT-qPCR	14	754		x
Exiqon	microRNA Ready-to-Use PCR panels	2	RT-qPCR	16	736	x	
	microRNA Ready-to-Use PCR panels	3	RT-qPCR	18	752	x	
	microRNA Ready-to-Use PCR panels	4	RT-qPCR	20	752	x	
	miRCURY LNA microRNA Array	10	microarray	10	722	x	
	miRCURY LNA microRNA Array	11	microarray	11	846	x	
	miRCURY LNA microRNA Array	5th gen	microarray	14	904	x	
	miRCURY LNA microRNA Array	6th gen	microarray	16	1218	x	
	miRCURY LNA microRNA Array	7th gen	microarray	20	2244	x	
Illumina	Human microRNA expression panel	1	microarray	9	470		x
Nanostring	nCounter Human miRNA panel	2	microarray	18	812	x	
	Nanostring NCounter Human	3	counter	21	829	x	
Qiagen	miScript miRNA PCR Arrays	16	RT-qPCR	16	1075		x
Quanta BioSciences	qScript microRNA System	1	microarray	20	2578	x	
Wafergen	SmartChip Human MicroRNA Panel	1	microarray	16	1046		x

Supplementary Figure 1

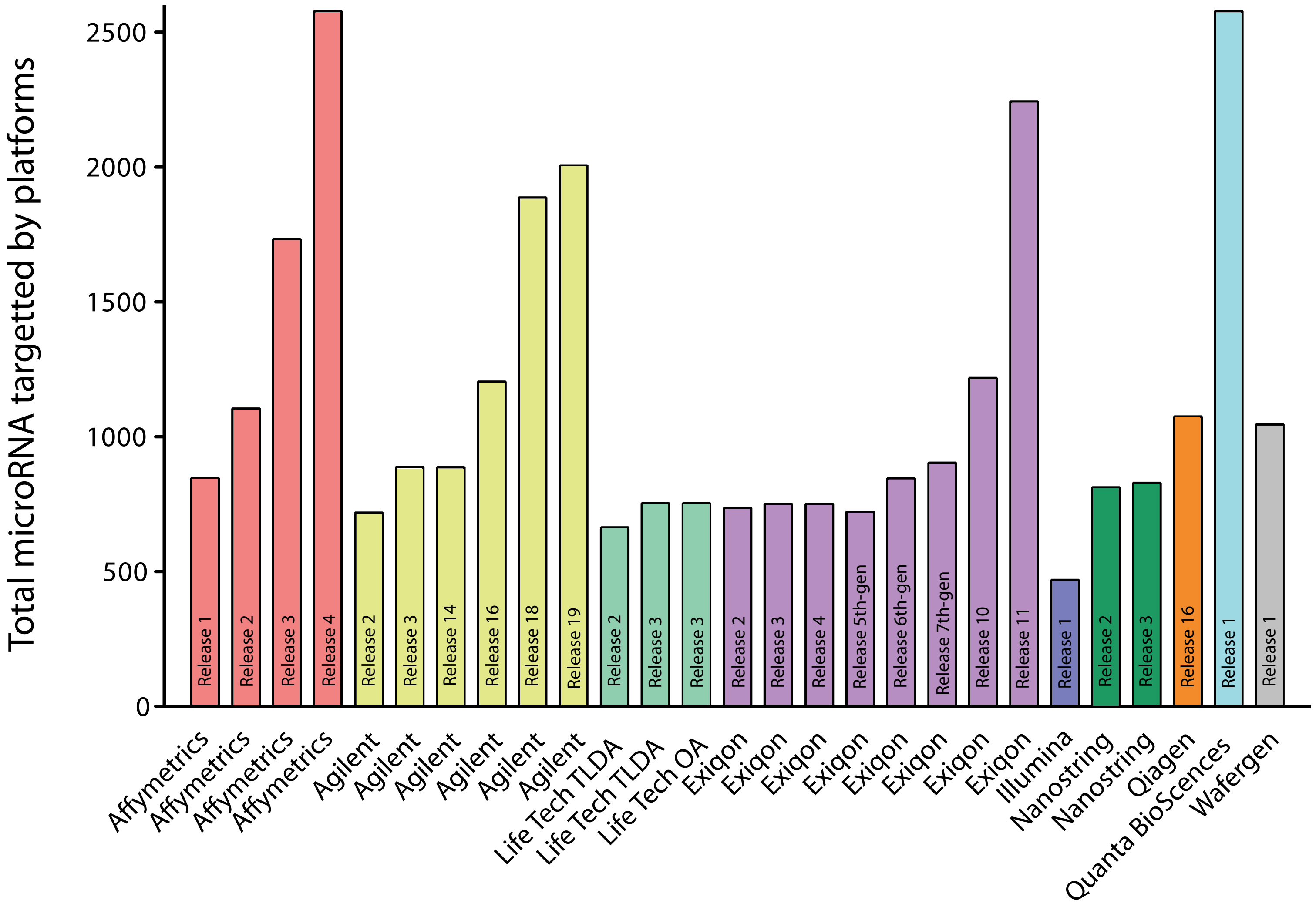

Supplementary Table 2

	Platform name	Release	Tecnology	miRBase	# miRNA	Seq by vendor	Seq by miRBase
Affymetrics	GeneChip miRNA Array mouse	2	microarray	15	717	x	
	GeneChip miRNA Array rat	2	microarray	15	387	x	
	GeneChip miRNA Array mouse	3	microarray	17	1111	x	
	GeneChip miRNA Array rat	3	microarray	17	679	x	
	GeneChip miRNA Array mouse	4	microarray	20	1908	x	
	GeneChip miRNA Array rat	4	microarray	20	728	x	
Quanta BioSciences	qScript microRNA System mouse	1	microarray	20	561		x
	qScript microRNA System rat	1	microarray	20	338		x
Exiqon	microRNA Ready-to-Use PCR panels Mouse	2	RT-qPCR	16	749	x	
	microRNA Ready-to-Use PCR panels Mouse	3	RT-qPCR	18	752	x	
	microRNA Ready-to-Use PCR panels Mouse	4	RT-qPCR	20	752	x	
LifeTech (Applied Biosystems)	TaqMan Array Rodent microRNA	3	RT-qPCR	14	750		x
	OpenArray Rodent microRNA	3	RT-qPCR	14	750		x

Supplementary Figure 2

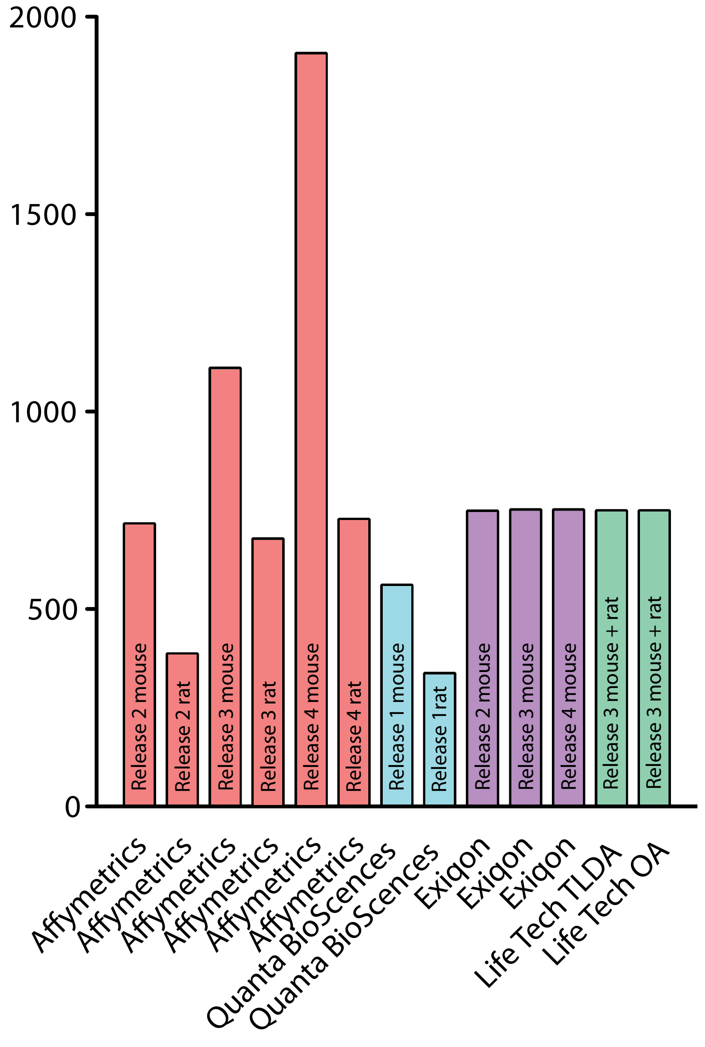

Supplementary Figure 3

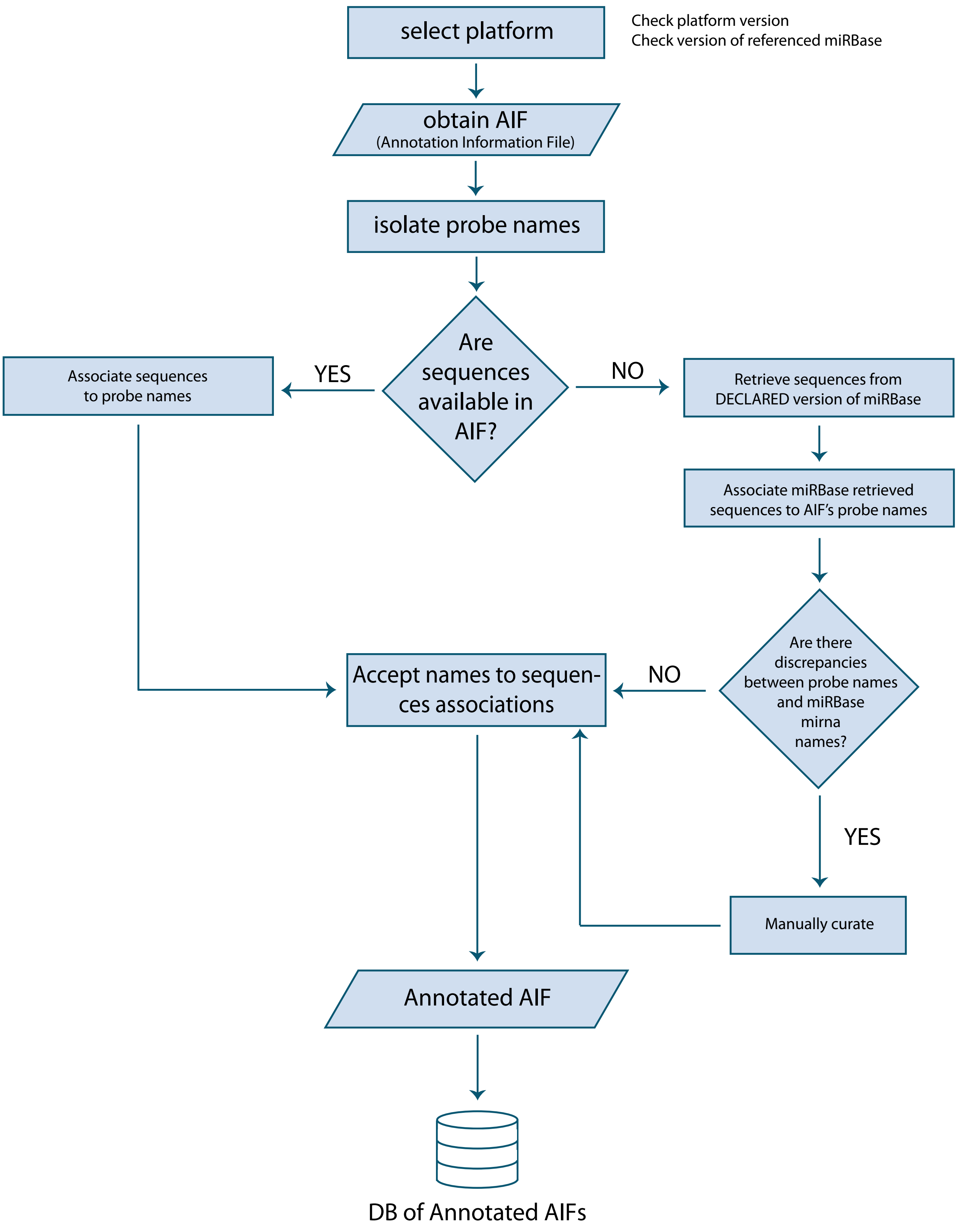

Supplementary Figure 4

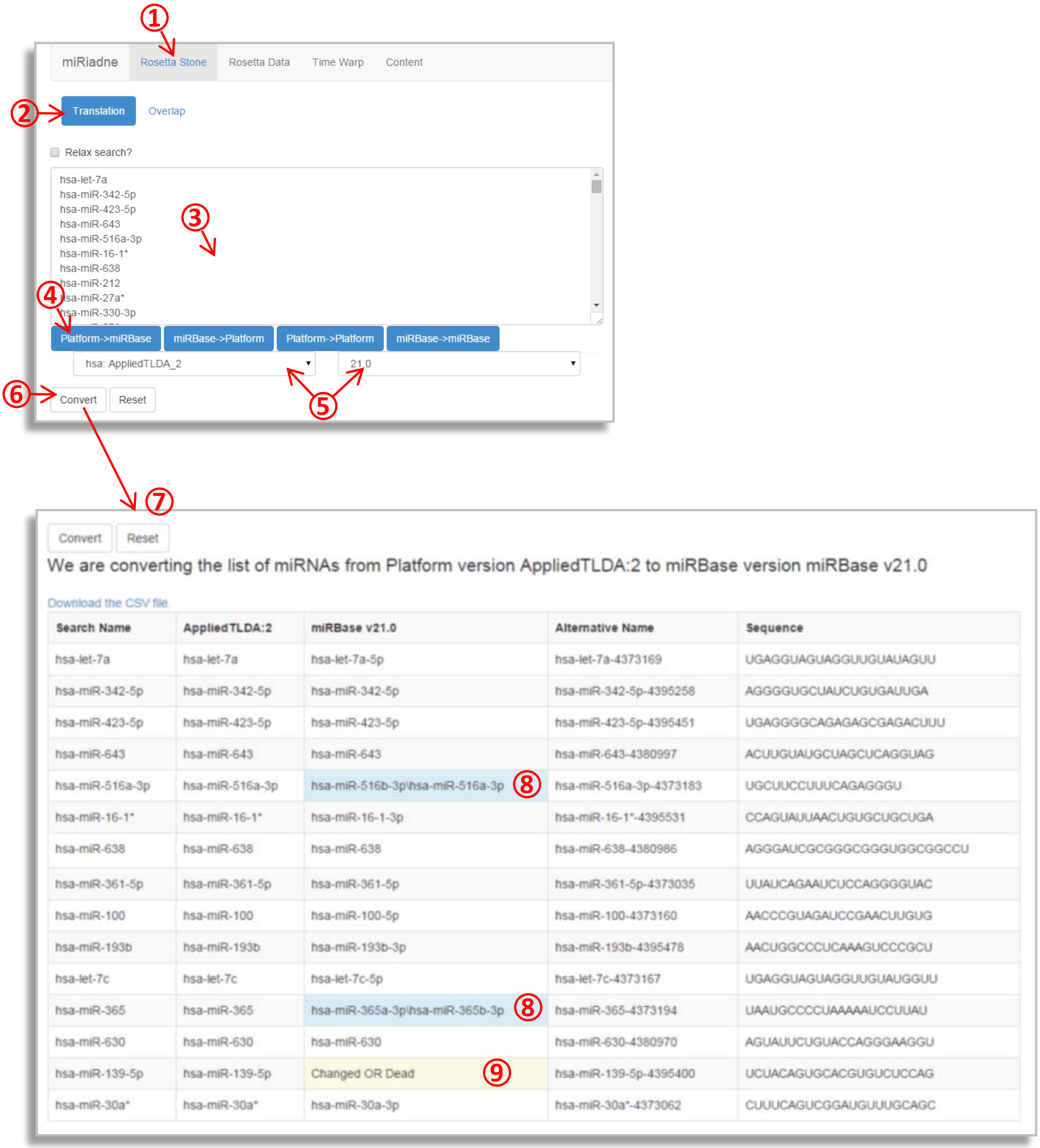

Search Name	AppliedTLDA:2	miRBase v21.0	Alternative Name	Sequence
hsa-let-7a	hsa-let-7a	hsa-let-7a-5p	hsa-let-7a-4373169	UGAGGUAGUAGGUUGUAUAGUU
hsa-miR-342-5p	hsa-miR-342-5p	hsa-miR-342-5p	hsa-miR-342-5p-4395258	AGGGGUGCUAUCUGUGAUUGA
hsa-miR-423-5p	hsa-miR-423-5p	hsa-miR-423-5p	hsa-miR-423-5p-4395451	UGAGGGGCAGAGAGCGAGACUUU
hsa-miR-643	hsa-miR-643	hsa-miR-643	hsa-miR-643-4380997	ACUUGUAUGCUAGCUCAGGUAG
hsa-miR-516a-3p	hsa-miR-516a-3p	hsa-miR-516b-3p\hsa-miR-516a-3p	hsa-miR-516a-3p-4373183	UGCUUCCUUUCAGAGGGU
hsa-miR-16-1*	hsa-miR-16-1*	hsa-miR-16-1-3p	hsa-miR-16-1*-4395531	CCAGUAUUAACUGUGCUGCUGA
hsa-miR-638	hsa-miR-638	hsa-miR-638	hsa-miR-638-4380986	AGGGAUCGCGGGCGGGUGGCGGCCU
hsa-miR-361-5p	hsa-miR-361-5p	hsa-miR-361-5p	hsa-miR-361-5p-4373035	UUAUCAGAAUCUCCAGGGGUAC
hsa-miR-100	hsa-miR-100	hsa-miR-100-5p	hsa-miR-100-4373160	AACCCGUAGAUCCGAACUUGUG
hsa-miR-193b	hsa-miR-193b	hsa-miR-193b-3p	hsa-miR-193b-4395478	AACUGGCCCUCAAAGUCCCGCU
hsa-let-7c	hsa-let-7c	hsa-let-7c-5p	hsa-let-7c-4373167	UGAGGUAGUAGGUUGUAUGGUU
hsa-miR-365	hsa-miR-365	hsa-miR-365a-3p\hsa-miR-365b-3p	hsa-miR-365-4373194	UAAUGCCCCUAAAAAUCCUUAU
hsa-miR-630	hsa-miR-630	hsa-miR-630	hsa-miR-630-4380970	AGUAUUCUGUACCAGGGAAGGU
hsa-miR-139-5p	hsa-miR-139-5p	Changed OR Dead	hsa-miR-139-5p-4395400	UCUACAGUGCACGUGUCUCCAG
hsa-miR-30a*	hsa-miR-30a*	hsa-miR-30a-3p	hsa-miR-30a*-4373062	CUUUCAGUCGGAUGUUUGCAGC

Supplementary Figure 5

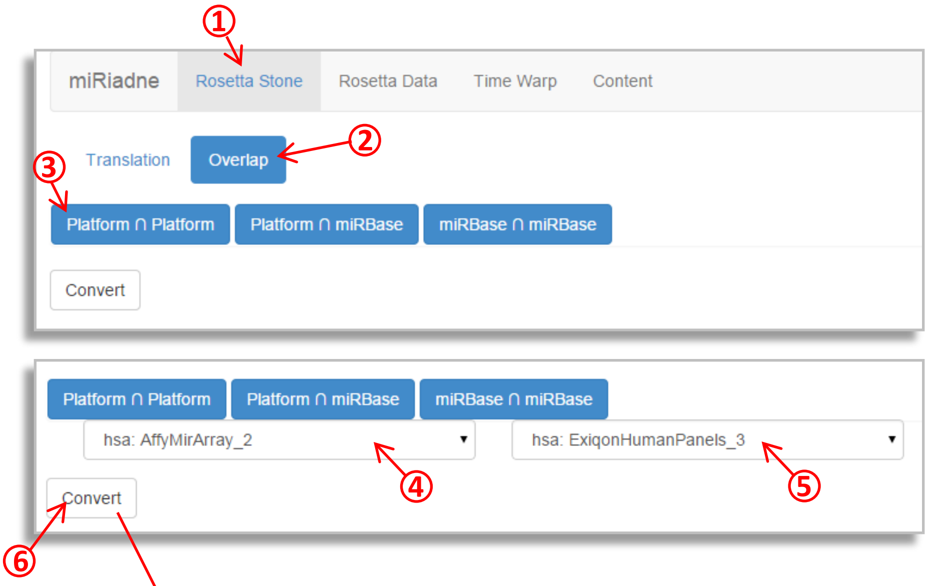

We are converting the list of miRNAs from Platform version AffyMirArray:2 to Platform version ExiqonHumanPanels:3

Search Name	AffyMirArray:2	ExiqonHumanPanels:3	Alternative Name	Sequence
hsa-let-7a	hsa-let-7a	hsa-let-7a-5p	hsa-let-7a-5p	UGAGGUAGUAGGUUGUAUAGUU
hsa-let-7a*	hsa-let-7a*	hsa-let-7a-3p	hsa-let-7a-3p	CUAUACAAUCUACUGUCUUUC
hsa-let-7a-2*	hsa-let-7a-2*	hsa-let-7a-2-3p	hsa-let-7a-2-3p	CUGUACAGCCUCCUAGCUUUCC
hsa-let-7b	hsa-let-7b	hsa-let-7b-5p	hsa-let-7b-5p	UGAGGUAGUAGGUUGUGUGGUU
hsa-let-7b*	hsa-let-7b*	hsa-let-7b-3p	hsa-let-7b-3p	CUAUACAACCUACUGCCUUCCC
hsa-let-7c	hsa-let-7c	hsa-let-7c	hsa-let-7c	UGAGGUAGUAGGUUGUAUGGUU
hsa-let-7d	hsa-let-7d	hsa-let-7d-5p	hsa-let-7d-5p	AGAGGUAGUAGGUUGCAUAGUU
hsa-let-7d*	hsa-let-7d*	hsa-let-7d-3p	hsa-let-7d-3p	CUAUACGACCUGCUGCCUUUCU
hsa-let-7e	hsa-let-7e	hsa-let-7e-5p	hsa-let-7e-5p	UGAGGUAGGAGGUUGUAUAGUU
hsa-let-7e*	hsa-let-7e*	hsa-let-7e-3p	hsa-let-7e-3p	CUAUACGGCCUCCUAGCUUUCC
hsa-let-7f	hsa-let-7f	hsa-let-7f-5p	hsa-let-7f-5p	UGAGGUAGUAGAUUGUAUAGUU
hsa-let-7f-1*	hsa-let-7f-1*	hsa-let-7f-1-3p	hsa-let-7f-1-3p	CUAUACAAUCUAUUGCCUUCCC
hsa-let-7f-2*	hsa-let-7f-2*	hsa-let-7f-2-3p	hsa-let-7f-2-3p	CUAUACAGUCUACUGUCUUUCC
hsa-let-7g	hsa-let-7g	hsa-let-7g-5p	hsa-let-7g-5p	UGAGGUAGUAGUUUGUACAGUU
hsa-let-7g*	hsa-let-7g*	hsa-let-7g-3p	hsa-let-7g-3p	CUGUACAGGCCACUGCCUUGC
hsa-let-7i	hsa-let-7i	hsa-let-7i-5p	hsa-let-7i-5p	UGAGGUAGUAGUUUGUGCUGUU
hsa-let-7i*	hsa-let-7i*	hsa-let-7i-3p	hsa-let-7i-3p	CUGCGCAAGCUACUGCCUUGCU
hsa-miR-1	hsa-miR-1	hsa-miR-1	hsa-miR-1	UGGAAUGUAAAGAAGUAUGUAU

Supplementary Figure 6

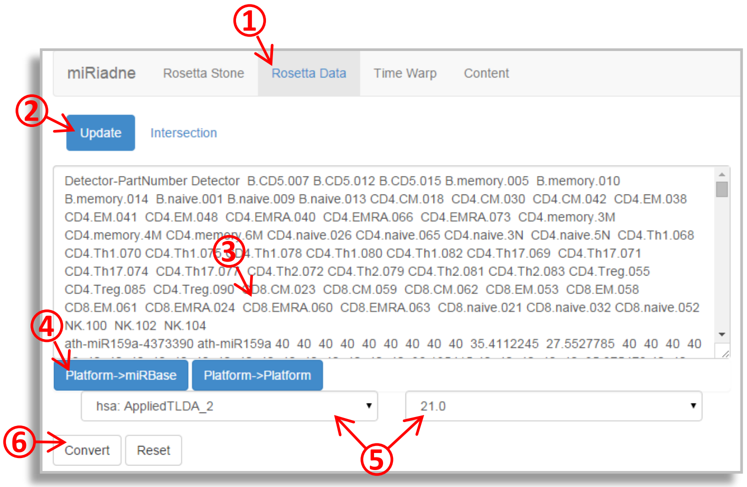

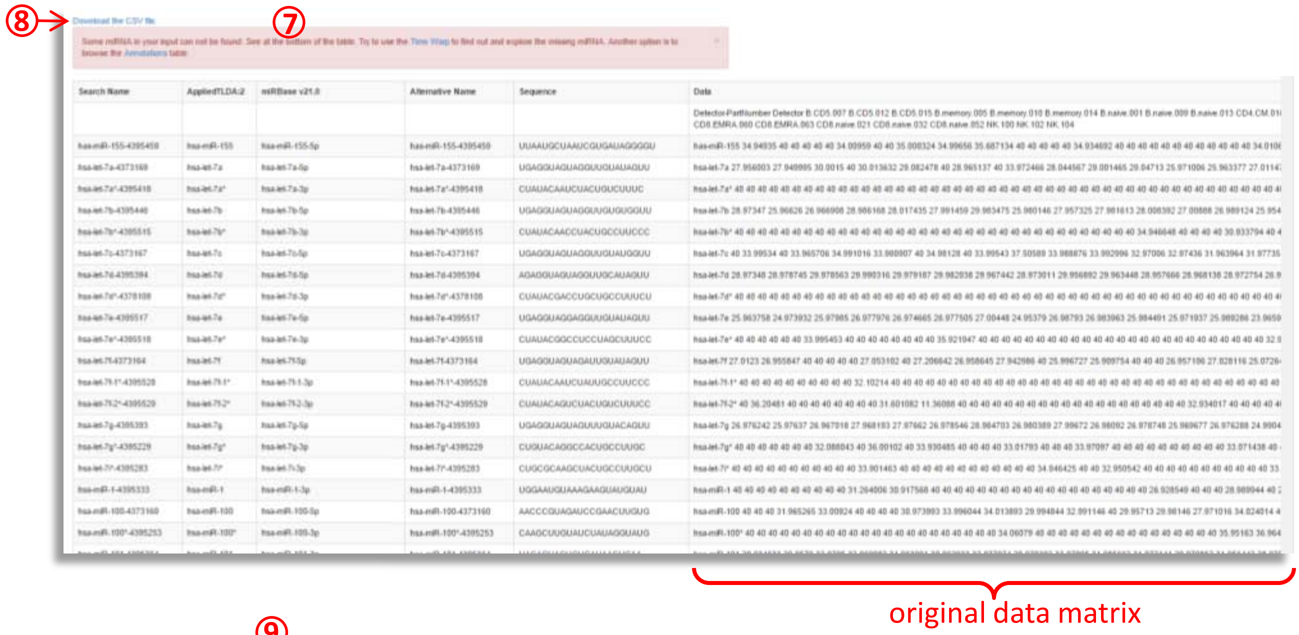

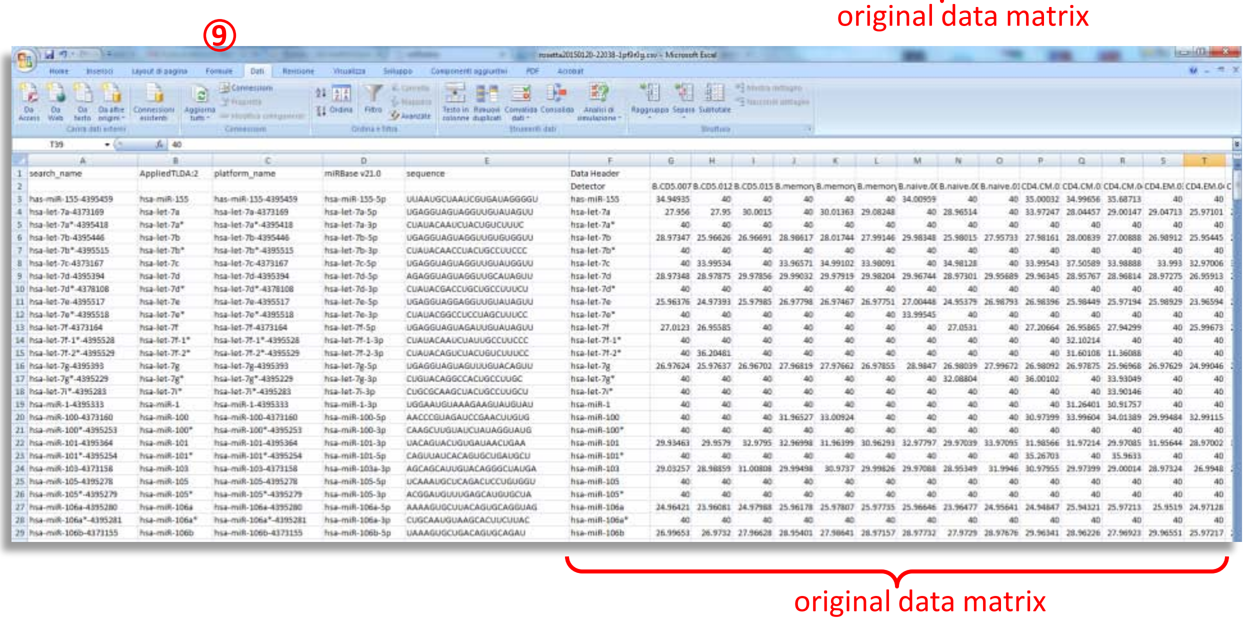

Supplementary Figure 7

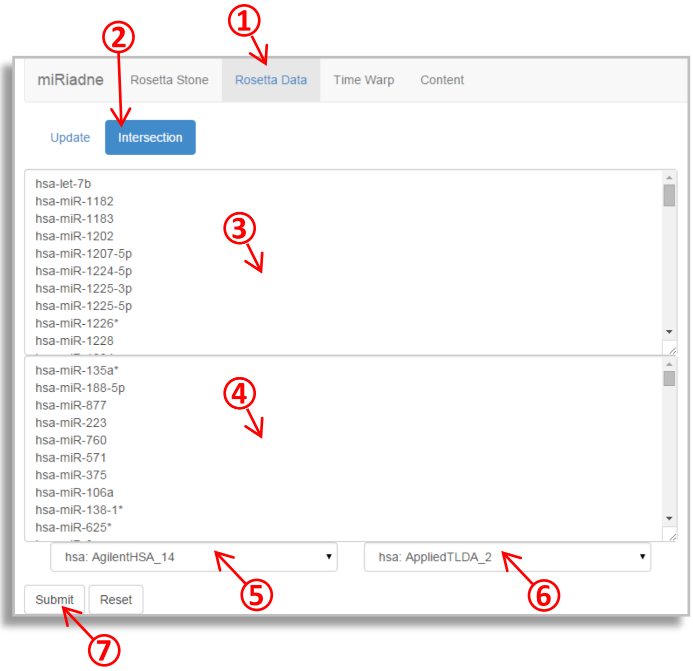

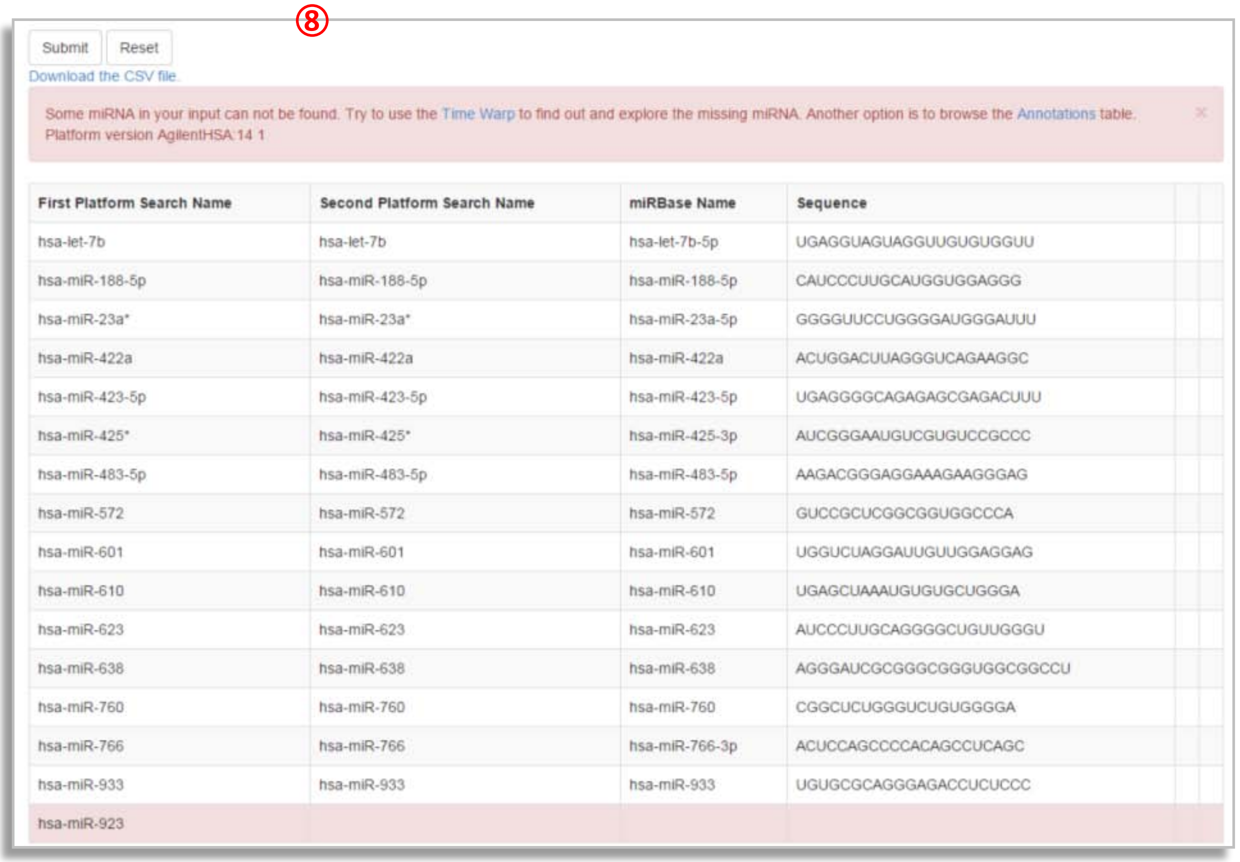

First Platform Search Name	Second Platform Search Name	miRBase Name	Sequence
hsa-let-7b	hsa-let-7b	hsa-let-7b-5p	UGAGGUAGUAGGUUGUGUGGUU
hsa-miR-188-5p	hsa-miR-188-5p	hsa-miR-188-5p	CAUCCCUUGCAUGGUGGAGGG
hsa-miR-23a*	hsa-miR-23a*	hsa-miR-23a-5p	GGGGUUCCUGGGGAUGGGAUUU
hsa-miR-422a	hsa-miR-422a	hsa-miR-422a	ACUGGACUUAGGGUCAGAAGGC
hsa-miR-423-5p	hsa-miR-423-5p	hsa-miR-423-5p	UGAGGGGCAGAGAGCGAGACUUU
hsa-miR-425*	hsa-miR-425*	hsa-miR-425-3p	AUCGGGAAUGUCGUGUCCGCCC
hsa-miR-483-5p	hsa-miR-483-5p	hsa-miR-483-5p	AAGACGGGAGGAAAGAAGGGAG
hsa-miR-572	hsa-miR-572	hsa-miR-572	GUCCGCUCGGCGGUGGCCCA
hsa-miR-601	hsa-miR-601	hsa-miR-601	UGGUCUAGGAUUGUUGGAGGAG
hsa-miR-610	hsa-miR-610	hsa-miR-610	UGAGCUAAAUGUGUGCUGGGA
hsa-miR-623	hsa-miR-623	hsa-miR-623	AUCCCUUGCAGGGGCUGUUGGGU
hsa-miR-638	hsa-miR-638	hsa-miR-638	AGGGAUCGCGGGCGGGUGGCGGCCU
hsa-miR-760	hsa-miR-760	hsa-miR-760	CGGCUCUGGGUCUGUGGGGA
hsa-miR-766	hsa-miR-766	hsa-miR-766-3p	ACUCCAGCCCCACAGCCUCAGC
hsa-miR-933	hsa-miR-933	hsa-miR-933	UGUGCGCAGGGAGACCUCUCCC
hsa-miR-923			

Supplementary Figure 8

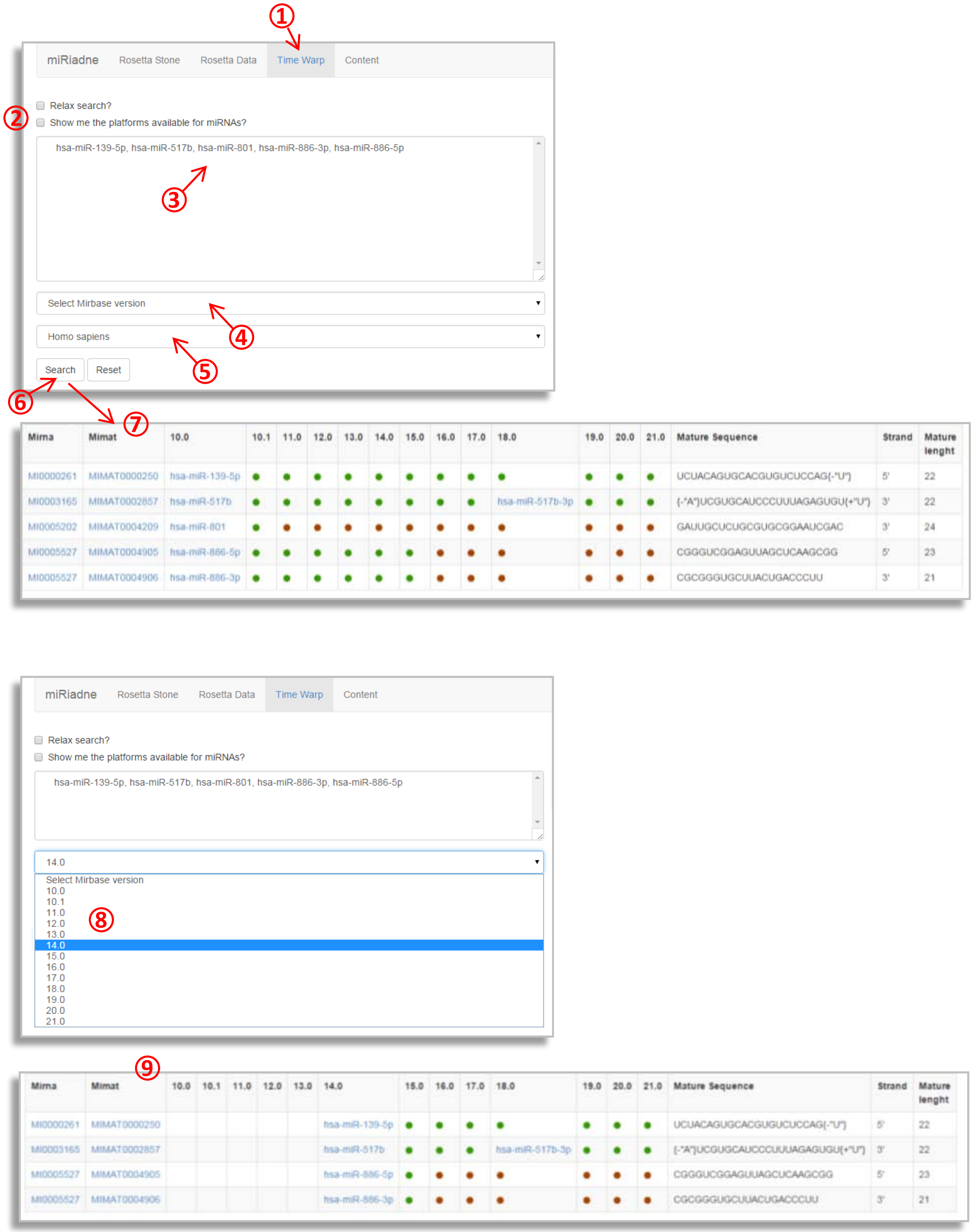

Supplement: SUPPLEMENTARY DATA [file supp_gkv381_nar-00249-web-b-2015-File004.pdf]
